# Effects of a school-based physical activity intervention on academic performance in 14-year old adolescents: a cluster randomized controlled trial – the School in Motion study

**DOI:** 10.1186/s12889-021-10901-x

**Published:** 2021-05-06

**Authors:** Runar Barstad Solberg, Jostein Steene-Johannessen, Sigmund Alfred Anderssen, Ulf Ekelund, Reidar Säfvenbom, Tommy Haugen, Sveinung Berntsen, Andreas Åvitsland, Øystein Lerum, Geir Kåre Resaland, Elin Kolle

**Affiliations:** 1 Department of Sports Medicine, Norwegian School of Sport Sciences, PB 4014, Ullevål Stadion, 0806 Oslo, Norway; 2 Faculty of Health and Sport Science, Department of Sport Science and Physical Education, University of Agder, PB 422, 4604 Kristiansand, Norway; 3 Department of Education and Sport Science, University of Stavanger, Pb 8600 Forus, 4036 Stavanger, Norway; 4 Department of Sport, Food and Natural Sciences, Faculty of Education, Arts and Sports, Western Norway University of Applied Sciences, Campus Sogndal, Box 133, 6851 Sogndal, Norway; 5 Center for Physically Active Learning, Faculty of Education, Arts and Sports, Western Norway University of Applied Sciences, Campus Sogndal, 6856 Sogndal, Norway

**Keywords:** Physical activity, Cluster RCT, Adolescents, Academic performance

## Abstract

**Background:**

School-based physical activity interventions evaluating the effect on academic performance usually includes children. We aimed to investigate the effect of a nine-month, school-based physical activity intervention titled School in Motion (ScIM) on academic performance in adolescents.

**Methods:**

Thirty secondary schools in Norway were cluster-randomized into three groups: the Physically active learning (PAL) group (*n* = 10), the Don’t worry – Be Happy (DWBH) group (*n* = 10) or control (*n* = 10). Target dose in both intervention groups was 120 min/week of additional PA during school hours. Parental consent was obtained from 2084 adolescent students (76%). Standardized national tests in reading and numeracy was conducted at baseline and at the end of the intervention. We used linear mixed model to test intervention effects. We found significant intervention effects in numeracy and reading among students in both interventions when compared with controls.

**Results:**

The mean difference in change in numeracy was 1.7 (95% CI: 0.9 to 2.5; Cohen’s d = 0.12) and 2.0 (95% CI: 1.4 to 2.7; Cohen’s d = 0.23) points in favour of students in the PAL and DWBH intervention, respectively. Similar results were found for reading, where the mean difference in change was 0.9 (95% CI 0.2 to 1.6; Cohen’s d = 0.06) and 1.1 (95% CI 0.3 to 1.9; Cohen’s d = 0.18) points in favour of students in the PAL and DWBH intervention, respectively. When conducting intention to treat analysis with imputed data the estimates were attenuated and some no longer significant.

**Conclusion:**

The ScIM study demonstrates that two different school-based PA interventions providing approximately 120 min of additional PA weekly over nine months, significantly improved numeracy and reading performance in 14-year old students compared with controls. However, the results should be interpreted with caution as the effect sizes reported were very small or small and the estimates were attenuated when conducting intention to treat analysis. Despite this, our results are still positive and suggest that PA interventions are viable models to increase academic performance among adolescents.

**Trial registration:**

Retrospectively registered (25/01/2019): NCT03817047.

## Background

Schools have received widespread attention owing to the inescapable pressure to educate students to meet accepted academic standards. Therefore, new effective teaching methods must be developed. Physical activity (PA) might be such a method because evidence has emerged concerning the positive association between PA and academic performance [1–6].

A randomized controlled trial (RCT) called Physical Activity Across the Curriculum, found improved performance in reading, numeracy, and spelling in a sub sample comparing children who received physically active lessons daily for three years with children who followed the regular curriculum [7]. Similarly, in the Fit & Vaardig op School study, weekly physically numeracy and language lessons over two years improved numeracy and spelling performance among elementary school children [8]. These results also correspond with the Activity and Motivation in Physical Education trail findings, where the authors reported significant intervention effects on numeracy performance [9].

Despite this, the literature is ambiguous as other school-based RCTs shows no intervention effect of multicomponent PA interventions on academic performance on whole group data among children [10, 11] and adolescents [12]. Finally, a recent systematic review on the effects of PA interventions on academic performance in 3–16 years olds concluded that strong evidence exist of the beneficial effects of PA on numeracy performance, but the evidence is inconclusive for overall academic performance [13].

Most studies investigating the effects of school-based PA interventions on academic performance are implemented among primary school children [7, 8, 10, 11]. Studies with adolescents have focused only on PE lessons [9] or have relatively short intervention period [12]. Consequently, few large, multicomponent PA interventions that include adolescents have been implemented over longer intervention periods in lower secondary school. Therefore, we conducted a school-based cluster RCT titled School in Motion (ScIM), consisting of two multicomponent PA interventions powered to compare changes in the mean PA level among secondary schools adolescents who received two extra hours of PA per week and a control group. We recently reported a favourable effect on the daily PA level and the time spent in moderate-to-vigorous intensity physical activity (MVPA) among adolescents in one of the intervention arms compared with controls [14]. In this paper, we investigated the intervention effect of the two PA interventions on academic performance in reading and numeracy.

## Methods

### Study design

The ScIM study was a nine-month school-based three-arm cluster RCT with schools as the cluster unit for randomization. The inclusion criteria were; > 25 students in ninth grade. We excluded schools that worked systematically with curriculum-prescribed PA and private and special schools. When a school agreed to attend, all students in the ninth grade were invited to participate. Four collaborating partners (Norwegian School of Sport Sciences, Western Norway University of Applied Sciences, University of Agder, and University of Stavanger) conducted the study during the 2017/2018 school year.

The ScIM study was reviewed and approved by the Norwegian Centre for Research Data and adhered to the Helsinki Declaration (2008). Parents or/guardians gave written informed consent allowing their child/ward to participate. The parents or adolescents could revoke this consent at any time. ScIM is registered in ClinicalTrials.gov (25/01/2019), ID nr: NCT03817047. The design, conduct, and reporting of this trial follow the recommendations of the CONSORT statement [15]. The CONSORT checklist is in related File 1.

### Randomization and blinding

Thirty schools were randomized manually by a lottery in a 1:1:1 ratio to either Physically active learning (PAL) intervention (*n* = 10), Don’t worry – Be Happy (DWBH) intervention (*n* = 10) or control (*n* = 10). One school withdrew after randomization but prior to baseline testing, leaving nine schools in the control group. The professional who performed the randomization did not partake in other parts of the study. Neither participants, schools, the testing personnel that performed the data collection in the schools nor researchers were blinded.

### The ScIM interventions

Both interventions aimed to provide approximately 120 min of additional PA in addition to the mandatory 120 to 180 min PE lessons per week. This goal was achieved by redistributing 5% of the other subjects to PA (60 min per week) and adding 60 min of PE to the curriculum. All intervention schools received $90 per student to account for the increased expenses.

The PAL-intervention focused on increasing student PA levels and consisted of three components (Table 1). We constructed an online toolkit of activities based on student and teacher feedback, and the existing pedagogical material that teachers could use. The PAL intervention was based on a socio-ecological theoretical framework that recognizes the complex interplay between personal and environmental influences on behaviour [16]. Teachers conducting the PAL intervention were encouraged to provide activities that would be enjoyable for all students.

**Table 1:** Intervention content and means of implementation stratified by intervention group.

Intervention components (min)	Practical organization	Providers of interventions	Implementation facilitation and method
Physically Active Learning (PAL)			
Physical activity in academic subjects (30 min)	Weekly	Teachers	An external collaborator provided a program tailored specifically to the subject curriculum. Teachers attended two courses during the intervention period.
Physical education (60 min)	Weekly	Physical education teachers	Follows the normal physical education curriculum
Physical activity (30 min)	Weekly	Teachers/physical education teachers	Students could choose between varied activities. Teachers were encouraged to motivate students during physical activity to stimulate their positive feelings and attitudes towards physical activity
Don’t Worry – Be Happy (DWBH)			
Activity class (Be happy class) (60 min)	Weekly	Teachers/physical education teachers	Self-organized activity developed according to the adolescent’s activity preferences.
Physical education (Don’t worry class) (60 min)	Weekly	Teachers/physical education teachers	Pupils led the regular PE class. Pupils practiced their Be Happy activity.

The focus of the DWBH-intervention was to promote friendship through PA, and it consisted of two components (Table 1): 60 min of a PE lesson called ‘Don’t worry’ (DW), and 60 min of a lesson called ‘Be happy’ (BH). First, students across different classes formed groups based on their interests. The groups performed the chosen activity in the BH lesson throughout the intervention period. The DW lessons were similar to an ordinary PE lesson, and the activities were either the same as in the BH lesson or were led by students representing one of the other activity groups in the BH lessons. The students developed the aims, management structure, strategies for impending conflicts, and routines for registration of attendance. This intervention was anchored to an integrative relational developmental system approach to human development, promoting mutually beneficial relationships for everyone involved [17].

At least one teacher from each intervention school attended a one-day course on how to deliver the intervention. The course consisted of theoretical and practical exercises by educators with experience in integrating PA into the curriculum. Control schools continued the current practice and did not implement additional curriculum-prescribed PA.

### Treatment group involvement

Teachers and students at the intervention schools were involved in the development of the two interventions in ScIM. Teachers and students were not involved designing the research questions, outcome measures, or analyses. The results of the study will be disseminated to all included schools.

### Delivery

All intervention components were mandatory. Adherence to intervention components was reported in an online platform. Each week, teachers at the intervention schools self-reported components performed or not performed, and the component intensity and minutes.

### Measurements

All measurements were obtained twice, first at baseline (April to August 2017) and second in the last phase of the intervention (April to June 2018). The test procedures were identical at both time points. The data were collected in the classroom and gymnasium. The research team trained all testing personnel, and all tests were conducted following the relevant guidelines.

### Academic performance

Academic performance was measured using standardized computer-based national tests designed and administered by The Norwegian Directorate for Education and Training. The numeracy test measured an individual’s ability to understand numbers and measurements. The reading test measured an individual’s basic Norwegian reading skills, interpreting and understanding texts, and considering the form and content. Both tests included anchor questions, making it possible to provide a baseline for an equating analysis between the two timepoints. The scores were standardized to a T-score with a mean of 50 scale points with a standard deviation (SD) of 10.

### Anthropometry

We measured the weight to the nearest 0.1 kg using a Seca 899 weight and measured the height to the nearest 0.1 cm using a SECA 123 Portable Stadiometer (SECA, Hamburg, Germany). We subtracted 0.6 kg (light clothing; gym shorts and t-shirt) or 1.5 kg (normal clothes; pants and sweater) from the weight measurements to account for clothing.

### Physical activity

We assessed PA using ActiGraph accelerometers, models GT3X and GT3X+ (ActiGraph, LLC, Pensacola, Florida, USA). Students were instructed to wear the accelerometer on their right hip over seven consecutive days, except when sleeping, showering and bathing. ActiLife software (ActiGraph, LLC, Pensacola, Florida, USA) was used to initialize and download the accelerometer files. Raw files were processed and analysed using STATA (Stata Statistical Software, StataCorp LP), and the epoch was 10 s. The data were recorded between 00:00 and 06:00, and all intervals of ≥20 consecutive min with no accelerations were excluded. Days with ≥480 min of active recording were considered valid. As a measure of the overall PA, we used average counts∙min^− 1^ (CPM) over the entire assessment period. To investigate the average minutes per day spent sedentary or in MVPA, we divided time registered with < 100 CPM and > 1999 CPM by the valid assessment days, respectively.

### Socioeconomic status

We linked our database to the registry data collected by Statistics Norway and used the highest education level of the participants’ parents as a proxy for socioeconomic status (SES). Four SES groups were computed low (primary/lower secondary/vocational high school), middle (secondary/high school), middle high (undergraduate degree), and high (graduate degree).

### Sample size

The ScIM study was powered to detect changes in the primary outcome (CPM) of 7% (49 CPM) between groups. The α level was 0.05 for all calculations. To detect a 49 ± 150 (mean ± SD) difference in CPM between the intervention groups and control group with a power of 0.9, expecting a dropout rate of 20%, we require at least 590 participants in each intervention arm.

### Statistics

In the main analysis, we included participants with a valid baseline or follow-up measures for academic performance. The data were assessed for normality and homogeneity of variance. The descriptive data are presented as the mean and SD unless otherwise stated. We fitted linear mixed models to both outcomes (numeracy and reading). Each model included fixed effects for the intervention, time (baseline and follow-up), and interaction term (intervention x time). As the units of randomization were schools, a “random effect” for school was included in the model, in addition to the class and subject ID to accommodate the clustering of students within these units.

We estimated the mean group values with a 95% confidence interval (CI) at the baseline and follow-up. We estimated the between group difference in change from the baseline to the follow-up between the participants in the intervention arms and control arm, adjusting for sex. Further, we examined whether sex modified the intervention effect by introducing an interaction term (timepoint x group x sex). A statistically significant interaction between sexes was evident in all academic performance models (*p* < 0.001 for interaction). Consequently, we repeated the analysis stratified by this variable.

We calculated a standardized mean difference score for each specific outcome (Cohen’s d), which was estimated using a random effects model. Cohen’s d values ranging from 0.01 to 0.20, 0.20 to 0.50 and 0.50 to 0.80 corresponds to very small, small and moderate effect size, respectively. We performed a per protocol analysis including schools with above 80% adherence to the protocol. Multiple imputations were performed on the academic performance variables as a sensitivity analysis to account for loss for the follow-up data. Imputation of variables was performed using chained equations (mi imputed chained) in Stata v.16. Mean differences in change, standard errors and 95% CI were obtained based on 20 imputed datasets. The imputation analyses assume that data are missing at random. All statistical analyses were performed using Stata (StataCorp LP).

## Results

**Fig. 1:**
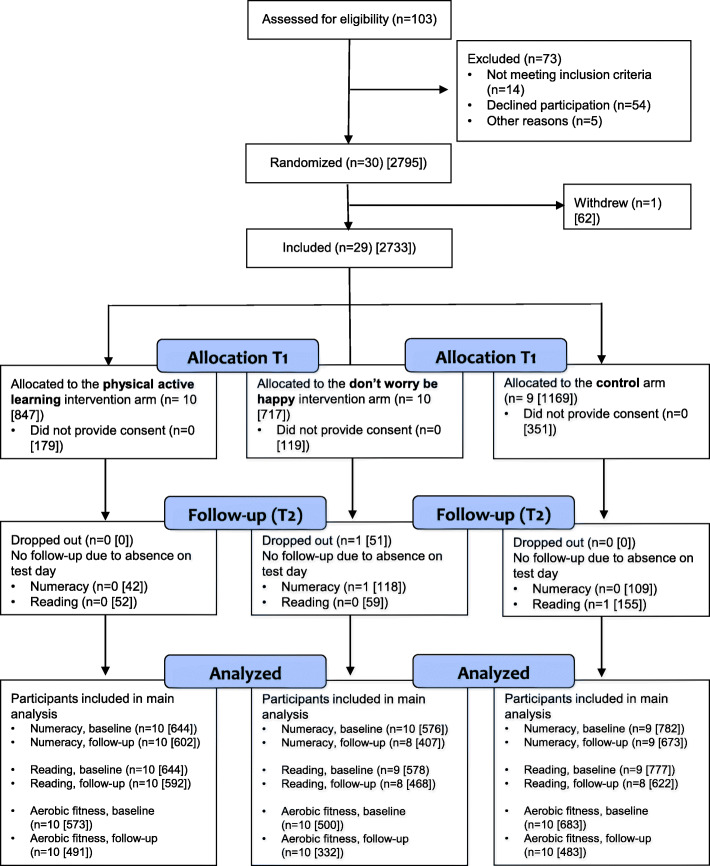
Flow of schools and students through the study. All numbers are schools [students]

The flow of the schools and participants is presented in Fig. 1. Among 2733 eligible students from 29 participating schools at baseline, parental consent was obtained from 2084 students (76%). One DWBH school withdrew from the trial after three months for practical reasons. At follow-up, one DWBH school was unable to complete the national test in numeracy and one control school were unable to complete the reading test. At baseline, 1999 and 2002 students had valid data on reading and numeracy respectively. A total of 1682 students had valid data in reading and numeracy at follow-up. The reason for the loss of follow-up data on academic performance was absences during the post-test (reading: *n* = 269; numeracy *n* = 266). The characteristics of all students by intervention group are presented in Table 2.

**Table 2:** Participant characteristics by group allocation at baseline. Mean (SD) unless other stated.

	PAL Intervention (*n* = 536–655)		DWBH Intervention (*n* = 427–586)		Control (*n* = 583–795)	
	Girls	Boys	Girls	Boys	Girls	Boys
N	328	327	286	300	387	408
Age (year)	13.9 (0.3)	13.9 (0.3)	13.9 (0.3)	14.0 (0.3)	14.0 (0.3)	14.0 (0.3)
**Parents education levels**						
Low (%)	5.8	7.0	5.2	7.6	2.5	7.1
Middle (%)	26.5	27.0	30.0	32.3	26.8	29.0
Middle high (%)	42.4	42.2	43.7	34.7	43.2	39.0
High (%)	23.8	22.9	20.2	24.3	26.1	23.5
**Anthropometry**						
Height (cm)	162.9 (6.2)	166.3 (9.4)	164.1 (6.1)	168.6 (8.4)	163.9 (6.5)	167.6 (8.3)
Weight (kg)	54.3 (9.6)	54.1 (11.8)	55.9 (10.2)	56.5 (11.7)	54.2 (9.3)	54.6 (11.5)
**Physical activity levels full day**						
Total PA (cpm)	473.2 (157.3)	552.1 (207.0)	512.8 (204.5)	563.7 (204.7)	510.5 (174.7)	590.1 (227.1)
MVPA (min/day)	64.5 (21.8)	71.6 (25.8)	69.6 (25.0)	73.3 (28.0)	69.7 (25.5)	77.8 (30.3)
Sedentary (min/day)	560.0 (69.9)	530.3 (86.7)	551.3 (75.4)	521.7 (80.6)	545.9 (72.0)	513.9 (82.9)
**Academic performance**						
Numeracy (points)	53.5 (9.8)	56.4 (9.8)	54.2 (9.3)	55.3 (9.4)	56.1 (9.9)	55.2 (10.1)
Reading (points)	56.5 (9.7)	54.2 (9.7)	55.8 (9.6)	53.0 (10.2)	57.5 (9.7)	52.7 (10.3)

PAL = Physical active learning; DWBH = Don’t worry – Be happy”. PA = physical activity; MVPA = moderate-to-vigorous intensity physical activity

### Effects on academic performance

**Fig. 2:**
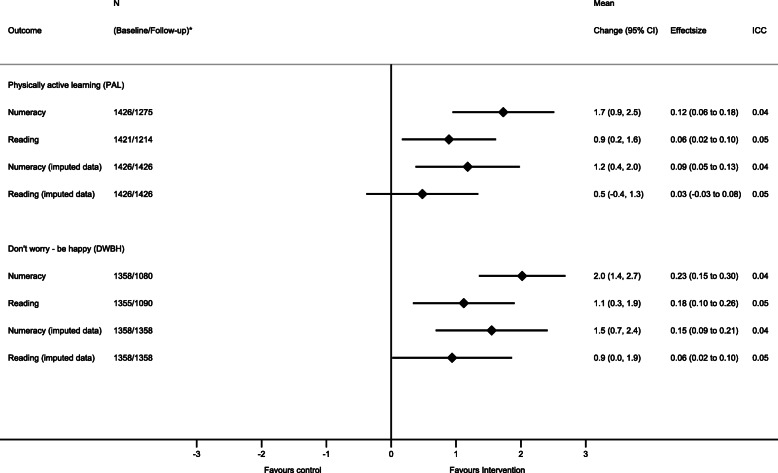
Intervention effect on academic performance in numeracy and reading stratified by study group compared with the control group. Each model contained fixed effects for intervention, time (baseline – follow-up), intervention x time interaction and random effects for school, class, and subject ID. CI = confidence interval; ICC = intraclass correlation coefficient (for school): N* indicates the number of participants in the analysis at baseline/follow-up in the intervention model and the control group

We found significant intervention effects on numeracy and reading among students in both interventions compared with the control group (Fig. 2). The mean difference in change in numeracy was 1.7 (95% CI: 0.9 to 2.5; Cohen’s d = 0.12) and 2.0 (95% CI: 1.4 to 2.7; Cohen’s d = 0.23) points in favour of students in the PAL and DWBH interventions, respectively. Similar results were found for reading, where the mean difference in change was 0.9 (95% CI 0.2 to 1.6; Cohen’s d = 0.06) and 1.1 (95% CI: 0.3 to 1.9; Cohen’s d = 0.18) points in favour of students in the PAL and DWBH interventions, respectively.

**Fig. 3:**
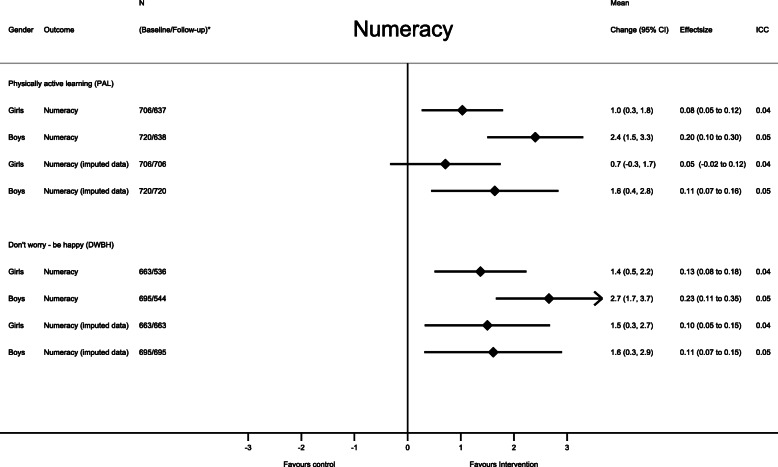
Intervention effect on academic performance in numeracy stratified by study group compared with the control group. Each model contained fixed effects for intervention, time (baseline – follow-up), intervention x time interaction and random effects for school, class, and subject ID. CI = confidence interval; ICC = intraclass correlation coefficient (for school): N* indicates the number of participants in the analysis at baseline/follow-up in the intervention model and the control group

**Fig. 4:**
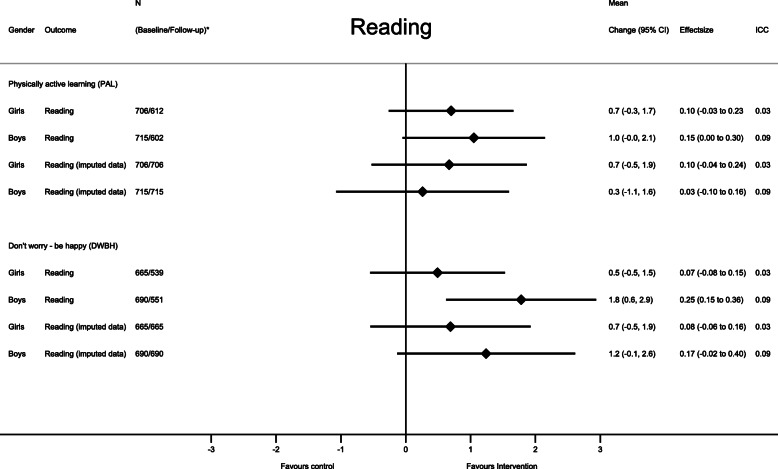
Intervention effect on academic performance in reading stratified by study group compared with the control group. Each model contained fixed effects for intervention, time (baseline – follow-up), intervention x time interaction and random effects for school, class, and subject ID. CI = confidence interval; ICC = intraclass correlation coefficient (for school): N* indicates the number of participants in the analysis at baseline/follow-up in the intervention model and the control group

Stratified by sex, the mean differences in change for numeracy in the PAL intervention were 1.0 points (95% CI: 0.3 to 1.8) and 2.4 points (95% CI: 1.5 to 3.3) for girls and boys respectively (Fig. 3). Similar findings were observed in the DWBH intervention, where the mean differences in change were 1.4 points (95% CI: 0.5 to 2.2) for girls and 2.7 points (95% CI: 1.7 to 3.7) for boys (Fig. 3). The mean difference in change in reading was attenuated when compared with the overall estimates. The only significant effect when the analysis was stratified by sex was among boys in the DWBH intervention compared with control group (Fig. 4).

### Sensitivity and per-protocol analysis

The sensitivity analysis from the imputed dataset followed the intention to treat (ITT) principle. Among students in the PAL-intervention, the mean difference in change in reading was attenuated to 0.5 points (95% CI: − 0.4 to 1.3; Cohen’s d = 0.03) (Fig. 2) and was no longer significant in the ITT-analysis. In the sex specific analysis, we found a similar pattern for numeracy among girls in the PAL intervention and for reading among boys in the DWBH intervention, where the estimates were attenuated and no longer significant when conducting the ITT-analysis (Figs. 3 and 4). The per-protocol analysis, including schools with a delivery rate of above 80%, did not differ from the main analysis (data not shown), except that the intervention did not show an intervention effect on numeracy performance among girls in the DWBH group when compared with control group (mean difference in change: 0.9, 95%CI: − 0.2 to 2.0).

### Intervention adherence

The adherence to the intervention protocol was 83 and 78% for PAL and DWBH interventions, respectively. Thus, the PAL-group delivered an average of 100 min/week of additional PA, and the DWBH-group delivered an average of 94 min/week of additional PA. The adherence varied between schools, ranging from 67 to 95%. Ten of the 19 intervention schools had a delivery rate of above 80%.

## Discussion

This paper aimed to evaluate the effect of two school-based PA interventions on academic performance among Norwegian adolescents. Both ScIM interventions resulted in better development over time in student academic performance in reading and numeracy than the control group.

Our findings are in line with recent intervention results suggesting a beneficial intervention effect of school-based PA on academic performance [7–9]. Although our results align with some studies, other studies do not support our findings [10–12]. The LCoMotion study included 632 Danish adolescents who performed 60 min of additional PA each school day over 20 weeks [12], The Active Smarter Kids study included 1100 Norwegian fifth graders who carried out 165 min of additional PA per week over seven months [11] and the Academic Achievement and Physical Activity Across the Curriculum study included 584 American children who engaged in more than 100 min of PA each week over three years [10]. The discrepancy in intervention effects could be due to several factors.

First, studies with non-significant results on academic performance consisted of various types of PA components, physically active learning, PA after school, active transportation and homework, short PA breaks during theoretical lessons, and recess [10–12]. Although no clear evidence indicate that some components of school-based PA would be more effective than others, Alvarez-Bueno et al. [18] concluded that all PA components, but especially PE, could improve academic performance. In ScIM, both interventions included additional PE, which LCoMotion, The Active Smarter Kids and Academic Achievement and Physical Activity Across the Curriculum study did not. However, neither did Physical Activity Across the Curriculum study [7] or the Fit & Vardig op School intervention [8], making it difficult to conclude which components of school-based PA are most effective.

Second, adherence to protocol might be of importance. In the LCoMotion [12], Academic Achievement and Physical Activity Across the Curriculum [10], and The Active Smarter Kids [11] studies, the teachers delivered an average of 40, 55, and 80%, respectively, of the weekly target dose across the intervention period. This adherence to protocol is lower than that reported in ScIM. When considering the per-protocol analyses it is important to emphasize that adherence was self-reported by the teachers every week across the intervention. Consequently, the self-report could be subject to bias; however, it is the same method used in the comparable studies. When designing and planning the ScIM interventions, we conducted a five-month pilot study, including seven schools and 700 adolescents, which led to adjusting both models to better reach the target group. The intervention models were simplified (i.e. one component – physically active breaks – was removed from the PAL model), and more resources were given to the teachers to increase adherence to the protocol. In ScIM, the teachers delivered approximately 80% of the intervention dose. The main reason for not reaching the intended target dose was various special events throughout the year (i.e., exams, holidays, and school trips). Nevertheless, ScIM indicates that it is possible to implement school-based PA interventions that positively affect academic performance in an already busy curriculum.

Third, the PAL intervention resulted in better daily PA development over time than the control group [14]. This results was also observed in the Physical Activity Across the Curriculum study [7], but was not reported in the other studies [11, 12]. The increased PA levels in the PAL model could theoretically be linked to changes in the brain structure, function or neurotransmitters concentration that occurs in students who are more physically active [19]. Furthermore, PA can affect the brain’s physiology by increasing the cerebral capillary growth, blood flow and nerve cells in the hippocampus, supporting learning and memory related to academic performance [19, 20].

In the DWBH intervention, we found effects on academic performance despite no effect on PA levels. However, the focus in the DWBH-intervention was to promote friendships through PA, which was more important than the dose and intensity of the activities. We speculate that an alternative explanation for the intervention effect on academic performance in the DWBH intervention is that the self-elected activities may have enhanced arousal, minimized fatigue and boredom, and led to higher levels of self-efficacy, which could optimize student academic performance [21]. Furthermore, the chosen activities may have encouraged students to cooperate with classmates, employ strategies and adapt to changing task demands. Studies have reported that PE enriched with social interaction improves inhibition [22, 23]. In addition, PA is associated with planning performance [24] and cognitive flexibility [25], which relates to better academic performance. Another possible mechanism is that varied PA through the curriculum enhances the enjoyment of academic lessons, leading to higher motivation and engagement with theoretical materials. This outcome can improve the classroom climate and subsequently act as a confounder for the intervention effect. However, when we rerun the analysis with adjustments for the classroom climate, the results did not change from the main analysis.

Our results suggest that the PA content and relational quality seems to be as important as the dose and intensity when aiming to increase students’ academic performance. These findings indicate that it is possible to develop new active teaching methods which could be more effective for increasing academic performance compared to more traditionally sedentary teaching methods. However, given limitations in small effect sizes and relative short intervention duration, more research is warranted. Studies implemented over a longer time period (e.g. two or three years) with direct measurement of cognition and other possible mechanisms can provide a more in depth understanding of how PA can affect academic performance among adolescents and should be prioritized.

The strengths of this study include the cluster randomized design using two different PA interventions, the high adherence to the protocols, and device measured PA, which ensures internal and external validity. Additionally, the large sample size (76% of eligible students) consisting of an understudied population recruited from four different regions across Norway reduces the risk of bias and suggests that the findings are generalizable to some extent. Finally, to provide an unbiased estimate of group allocation, we performed a mixed model analysis with all participants with valid data on either time point for academic performance. Multiple imputation when performing mixed models analyses can lead to unstable results [26], and when the analysis was rerun on the imputed data the estimates were attenuated and some no longer significant.

We did not include any measurement of cognition or biological pathways on how PA might influence academic performance. Furthermore, although several studies have used national tests to measure academic performance, these could be a potential limitation because no validation studies exist. Another limitation is that the effect size (Cohen’s d) of the intervention effect in the primary analysis is considered very small or small (d = 0.06 to 0.23). However, other intervention studies that have demonstrated beneficial effects on academic performance have been implemented over two school years. As our intervention only lasted for nine months, we can speculate regarding whether a longer intervention duration would result in further improvements in academic performance.

## Conclusion

The ScIM study demonstrates that two different school-based PA interventions providing approximately 120 min of additional PA weekly over nine months, significantly improved numeracy and reading performance in 14-year old students compared with controls. However, the results should be interpreted with caution as the effect sizes reported were very small or small and the estimates were attenuated when conducting ITT analysis. Despite this, our results are still positive and suggest that PA interventions are viable models to increase academic performance among adolescents.

## Data Availability

The datasets generated and/or analyzed during the current study are not publicly available as publications are planned but are available from the corresponding author on reasonable request.

## Abbreviations

DWBH: Don’t worry – be happy
MVPA: Moderate-to-vigorous intensity physical activity
PA: Physical activity
PAL: Physically active learning
PE: Physical education
RCT: Randomized controlled trial
ScIM: School in motion
